# Typhoid fever: the challenging diagnosis of a pseudo-outbreak in Benguet, Philippines

**DOI:** 10.5365/wpsar.2024.15.3.1047

**Published:** 2024-07-12

**Authors:** Jorah May C Guzman, Ray Justin C Ventura, Maria Zheila C Blanco, Karen B Lonogan, Rio L Magpantay

**Affiliations:** a Field Epidemiology Training Program–Intermediate Course; Provincial Health Office, Benguet, Philippines.; b Department of Health, Manila, Philippines.

## Abstract

**Objective:**

The event-based surveillance and response report from the municipality of Buguias in the Philippines covering the period 1 January to 29 October 2022 indicated an unusual increase in the number of typhoid cases that surpassed the epidemic threshold for consecutive weeks. An investigation was conducted to confirm the existence of an outbreak, identify the source(s) of transmission and recommend prevention and control measures.

**Methods:**

The investigation employed a descriptive design. Medical records were reviewed to verify diagnoses and to identify cases that met case definitions. Key informant interviews were conducted to identify possible sources of transmission and investigate the reporting of cases in the Philippine Integrated Disease Surveillance and Response (PIDSR) system.

**Results:**

A total of 220 cases of typhoid fever were captured by the PIDSR system. Of the 208 suspected cases that were reviewed, only 15 (7.2%) met the case definition used in this investigation. Fourteen of these 15 verified cases were interviewed; five (35.7%) were farmers and 13 (92.8%) reported using springs as their main water source and source of drinking-water. Reporting of cases in the PIDSR system was largely based on the final chart diagnosis or a positive Typhidot or Tubex rapid diagnostic test result. The PIDSR case definition was not followed in the reporting of cases.

**Discussion:**

This study provides evidence of endemicity of typhoid fever in Buguias, Benguet, Philippines. However, from January to October 2022, cases were overreported by the surveillance system. Medical record reviews showed that most reported suspected cases did not meet case definition criteria. This finding emphasizes the need to improve typhoid guidelines with regards to diagnosis using rapid diagnostic tests and to investigate the cost–effectiveness of making confirmatory laboratory tests for typhoid available in the Philippines.

Between 1 January and 29 October 2022, the Philippine Integrated Disease Surveillance and Response (PIDSR) system reported a total of 9057 cases of typhoid fever. (*1*) This was a 121% increase in case numbers relative to the same period in 2021, when 4102 cases were reported. The Cordillera Administrative Region reported the highest number of cases, with Benguet Province being particularly affected. Benguet Province reported 1196 cases, accounting for 66% of all cases in the Cordillera region. (*1*)

Typhoid fever is caused by the bacterium *Salmonella typhi*, of which humans are the only reservoir. Other serotypes, such as *Salmonella paratyphi* (A, B, C), cause similar syndromes but are associated with less clinically significant disease and have an animal reservoir. (*2*) Salmonella is commonly found in poultry products, meats and chicken manure. Reusing chicken litter exacerbates contamination, especially with Salmonella. (*3*) Flies can also transmit typhoid, carrying bacteria and viruses in their vomit and excreta. (*4*) Inadequate sanitation and reliance on unsafe drinking-water sources can also contribute to the spread of typhoid. (*5*)

According to a routine event-based surveillance and response report from the Buguias Municipal Health Office Epidemiology and Surveillance Unit (unpublished), in 2022, Buguias, a municipality in Benguet Province, experienced an unusual increase in the number of typhoid cases that surpassed the Philippines’ standard epidemic threshold for several consecutive weeks. Buguias, with a total population of 44 877 (as of 2020), (*6*) is primarily an agricultural town known for its production of highland vegetables such as lettuce, cabbage, carrots and broccoli. (*7*) An outbreak investigation was conducted to confirm the existence of the outbreak and verify the cases, identify the source of transmission, and recommend prevention and control measures.

## Methods

This study employed a retrospective case review design to investigate the increase in the number of typhoid fever cases reported to the surveillance system in Buguias, Benguet from 1 January to 29 October 2022 (epidemic weeks 1–43; the study could not encompass the entire year due to the limited length of the authors’ training programme). Medical records of cases captured by the PIDSR system were obtained from local hospitals and reviewed to assess whether they met the PIDSR case definition and a slightly modified version of the PIDSR case definition. The modified version was used in this investigation to avoid missing cases that do not meet the stricter PIDSR definition, as not all symptoms were captured in the medical records and most cases could not be interviewed retrospectively. The PIDSR defined a suspected case as any person with an illness characterized by insidious onset of sustained fever, headache, malaise, anorexia, relative bradycardia, constipation or diarrhoea, and non-productive cough. The slightly modified version defined a suspected case as any resident of Buguias, Benguet who developed symptoms including fever, headache, malaise and any one or more of anorexia, constipation, diarrhoea, non-productive cough, vomiting, abdominal pain or dizziness, and who had a positive rapid diagnostic test result (Typhidot or Tubex) during the specified period.

Key informant interviews were conducted with Municipal Epidemiological Surveillance Unit staff and sanitary inspectors to identify possible sources of transmission and to discuss the management of the rise in cases of typhoid fever in the municipality. Hospital Epidemiology and Surveillance Unit staff were also interviewed as part of the investigation of the rise in case numbers reported by the PIDSR system. Informed consent was obtained from all respondents.

Suspected cases that met the modified case definition were interviewed using a structured questionnaire designed to collect information about water sources and treatment, as well as food preparation and storage practices. All cases gave their informed consent for the interview. In addition, the houses and immediate surroundings of each interviewed case were inspected and environmental samples (e.g. water, chicken manure, raw beef) were collected for laboratory testing. The latter was conducted using Salmonella Shigella Agar, a selective and differential medium for the isolation and enumeration of Salmonella and Shigella by means of the direct plating method. After inoculation of samples on the agar, plates were incubated for 48–72 hours before screening. Positive cultures showing black centre colonies were subjected to Phoenix automated identification and antibiotic sensitivity tests to confirm the presence of bacterial pathogens.

A profile of each suspected case was created and encoded in Microsoft Excel. Key characteristics of cases were summarized using descriptive statistics: categorical variables (such as sex) were described in terms of proportions, and continuous variables (such as age) were reported using measures of central tendency and dispersion (median and range). Weekly case numbers were compared with pre-defined alert and epidemic thresholds, which were derived from disease incidence data collected over the previous 5 years and calculated using the standard deviation method.

## Results

### Medical records review

The PIDSR system recorded 220 suspected cases of typhoid fever during the period 1 January–29 October 2022 for the municipality of Buguias, of which 208 (94.5%) were reviewed using medical records to verify the diagnosis. Of the reviewed cases, 178 (85.6%) had less than four symptoms, and 29 (13.9%) had only one symptom. Only 15 (7.2%) cases met the modified case definition criteria used in this study, while none met the PIDSR case definition.

Of the 205 discarded cases (i.e. cases that did not meet the case definition criteria), 198 (96.6%) had a positive serological test result, either through Typhidot (*n* = 177, 86.3%) or Tubex (*n* = 21, 10.2%). About half (*n* = 104, 50.7%) had an additional diagnosis other than typhoid fever such as acute gastroenteritis (*n* = 31, 15.1%), respiratory tract infection (*n* = 30, 14.6%) or dengue (*n* = 27, 13.2%). In terms of the symptoms presented, 77 (37.6%) did not manifest fever, while 16 (7.8%) only had fever as a symptom (**Table 1**).

**Table 1:** Characteristics of false positive typhoid cases reported to the surveillance system, Municipality of Buguias, the Philippines, 1 January–29 October 2022 (*n* = 205)

Characteristics	No. of cases	% of total
*Serological test*		
Positive Typhidot	177	86.3
Positive Tubex	21	10.2
Negative	4	2.0
Not done	3	1.5
*Final diagnosis in medical chart*		
Typhoid fever only	89	43.4
Typhoid fever and acute gastroenteritis	31	15.1
Typhoid fever and upper/lower respiratory tract infection	30	14.6
Typhoid fever and dengue	27	13.2
Typhoid fever and other	16	7.8
None	12	5.9
*Symptom profile*		
No fever	77	37.6
Fever plus 2 other typhoid-like symptoms	45	22.0
Fever plus 1 other typhoid-like symptom	29	14.1
Fever plus cough only	22	10.7
Fever only	16	7.8
Fever plus vomiting only	9	4.4
Fever plus 3 other typhoid-like symptoms	7	3.4

**Fig. 1:**
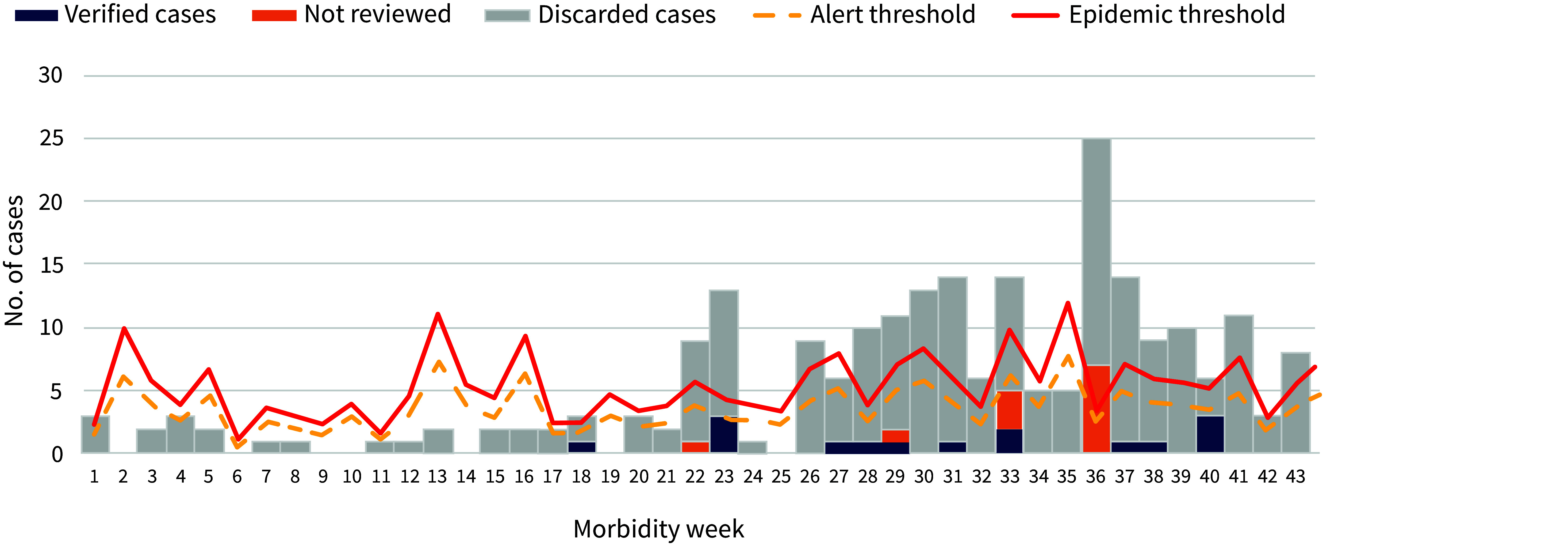
Typhoid cases by morbidity week, Municipality of Buguias, the Philippines, 1 January–29 October 2022 (N = 220)

When the discarded cases were excluded, the number of typhoid cases breached the alert threshold only once (in morbidity week 23). In comparison, when all 220 recorded cases were considered, i.e. including the false positives that did not meet the modified case definition, the epidemic threshold was breached in 15 out of the 43 morbidity weeks covered by this study (**Fig. 1**).

[**insert** Figure 1]

### Profile of cases

Of the 15 verified cases, just over half were male (*n* = 8, 53.3%). Cases ranged in age from 14–66 years, with a median age of 31 years. The most common age group was 21–30 years (*n* = 5, 33.3%). Fourteen verified cases (93.3%) were interviewed. One third of the interviewed cases were farmers (*n* = 5, 35.7%); all 14 said that they lived within 50 m of a farm and 13 reported relying on springs as the main source of their drinking-water (92.9%).

Seven cases (46.7%) reported that, in addition to fever, headache and malaise, they had experienced non-productive cough; five (33.3%) also experienced dizziness. Among the interviewed cases, all 14 reported that they normally wash their hands before handling food. Nine (64.3%) said that they sometimes ate raw vegetables. Most reported eating eggs at least once a month (*n* = 9, 64.3%), but none ate raw or soft-boiled eggs. Beef and chicken were seldom consumed by the majority of those interviewed (*n* = 13, 92.9%); only one person consumed beef and chicken regularly and none consumed raw meat. All 14 interviewed cases reported always seeing houseflies in their houses and self-reported that flies frequently land on their food.

### Key informant interviews

According to the sanitary inspector, springs are the main source of water for many households living in and around Buguias. It is also the most common source of drinking-water among farming communities. However, spring water is not always treated before use by some of the population as they believe it is clean and safe to drink. The sanitary inspector identified chicken manure as a possible source of water contamination, noting that compliance with an ordinance mandating that farmers who use chicken manure keep it in a storage area is not currently being adequately monitored.

### Environmental investigation

Across the municipality, chicken manure is widely used as a fertilizer by farmers and thus flies are commonplace. Locally reared livestock products (beef, chicken and pork) are sold within the community, typically from open, uncovered displays, upon which flies are free to land.

Of the 12 water samples that were taken from domestic taps and water storage tanks and tested, two showed the presence of black colony formation. One out of six chicken manure samples showed the presence of black colony formation. One sample of raw beef taken from a beef store also showed the presence of black colony formation. Black colonies from a sample of chicken manure fertilizer were subjected to a confirmatory culture and sensitivity test and tested positive for *Leminorella grimontii*. No Salmonella species were detected. Confirmatory testing was negative for all other samples with black colony formation.

## Discussion

Although 220 suspected cases of typhoid fever were reported in the municipality of Buguias, Benguet between January and October 2022, this investigation showed that most of them did not meet the modified case definition. Moreover, the pattern of case reporting revealed that the disease reporting units relied heavily on the diagnosis recorded in the medical chart and the results of Typhidot and Tubex rapid diagnostic testing. While Typhidot is a sensitive test for early diagnosis of typhoid fever, it has low specificity, and positive results should be correlated with the clinical picture and other possible diagnoses. Other studies have demonstrated that cross-reactivity with other diseases can cause false-positive results in some cases. (*8*) After verifying the reported diagnoses, we found that the number of cases was aligned with historical surveillance data and therefore we concluded that typhoid cases had been overreported and that the increase in case numbers in 2022 represented a pseudo-outbreak.

Although the number of verified cases was above the alert threshold in morbidity week 23, this increase is not inconsistent with, and generally follows, the endemic pattern. Routine surveillance has shown that there has been steady but low-level transmission of typhoid fever over a 5-year period and that known sources of transmission, including houseflies (*4*, *9*) and consumption of untreated drinking-water, (*5*) are likely present in the municipality. It is notable that all the verified cases self-reported a constant presence of houseflies in their houses, and that their food was frequently exposed to flies. While this implies that the opportunity for flies to transmit the bacteria that cause typhoid fever does indeed exist in this community, the findings do not prove a causal relationship between houseflies and typhoid fever.

This investigation also revealed that a high proportion of the verified cases relied on untreated spring water as their main source of drinking-water. The use of unsafe drinking-water has been linked to an increased rate of typhoid fever in other studies, (*5*) and may be another possible contributory factor for disease transmission in the municipality. This observation highlights the need for proper water treatment and quality checks to ensure that the water supply is safe for consumption and to prevent the spread of waterborne diseases including typhoid.

The main limitation of this study is the lack of confirmatory laboratory tests, as it is not the standard to carry out these tests during clinical encounters in the Philippines, and we could not conduct them retrospectively. However, based on the clinical presentation of the suspected cases and the epidemiological context, we believe that the 205 cases we excluded were likely false positives.

Reporting false positive cases can result in false outbreaks being declared and the misallocation of resources to unnecessary response efforts. To prevent this, it is crucial to enhance adherence to PIDSR protocols by providing disease surveillance officers with training in case assessment to ensure cases meet the case definition before they are included in the surveillance system report and by conducting regular evaluations of the surveillance system. Given the apparent reliance on rapid diagnostic test results for typhoid, we also recommend that diagnostic algorithms be used with clinical judgement and appropriate follow-up testing to ensure that fewer false positives are reported by the PIDSR system. Finally, this study emphasizes the need to improve typhoid guidelines with regards to diagnosis using rapid diagnostic tests and to investigate the cost–effectiveness of making confirmatory laboratory tests for typhoid available in the Philippines.
